# Friend versus foe: Neural correlates of prosocial decisions for liked and disliked peers

**DOI:** 10.3758/s13415-017-0557-1

**Published:** 2018-01-09

**Authors:** Elisabeth Schreuders, Eduard T. Klapwijk, Geert-Jan Will, Berna Güroğlu

**Affiliations:** 1 Institute of Psychology, Leiden University, Leiden, The Netherlands; 2 Leiden Institute for Brain and Cognition (LIBC), Leiden, The Netherlands; 3 Child and Adolescent Psychiatry, Curium-Leiden University Medical Centre, Leiden, The Netherlands; 4 Max Planck UCL Centre for Computational Psychiatry and Ageing Research, University College London, London, UK

**Keywords:** Peer relationships, Social decision-making, fMRI, Prosocial behavior, Dictator game

## Abstract

Although the majority of our social interactions are with people we know, few studies have investigated the neural correlates of sharing valuable resources with familiar others. Using an ecologically valid research paradigm, this functional magnetic resonance imaging study examined the neural correlates of prosocial and selfish behavior in interactions with real-life friends and disliked peers in young adults. Participants (*N* = 27) distributed coins between themselves and another person, where they could make selfish choices that maximized their own gains or prosocial choices that maximized outcomes of the other. Participants were more prosocial toward friends and more selfish toward disliked peers. Individual prosociality levels toward friends were associated negatively with supplementary motor area and anterior insula activity. Further preliminary analyses showed that prosocial decisions involving friends were associated with heightened activity in the bilateral posterior temporoparietal junction, and selfish decisions involving disliked peers were associated with heightened superior temporal sulcus activity, which are brain regions consistently shown to be involved in mentalizing and perspective taking in prior studies. Further, activation of the putamen was observed during prosocial choices involving friends and selfish choices involving disliked peers. These findings provide insights into the modulation of neural processes that underlie prosocial behavior as a function of a positive or negative relationship with the interaction partner.

**Electronic supplementary material:**

The online version of this article (10.3758/s13415-017-0557-1) contains supplementary material, which is available to authorized users.

Throughout the day, we interact with all kinds of people, such as people we know and strangers. The majority of our interactions are most likely to involve liked others, such as friends, but sometimes they involve those we do not like. Friends provide support and company (Hartup, 1996), whereas relationships based on dislike are characterized by aggression, attempts to do harm, and avoidance (Card, 2007). It is therefore not surprising that individuals tend to behave in a more prosocial manner toward friends than toward disliked peers (Güroğlu, van den Bos, & Crone, 2014a). Moreover, prosocial behaviors that maximize outcomes for the other person are important for forming and maintaining friendships (Eisenberg, Fabes, & Spinrad, 2006; Fehr, Fischbacher, & Gächter, 2002), whereas nonprosocial or selfish behaviors that maximize outcomes for the self may weaken a relationship and may even provide a basis for relationships based on dislike. A better understanding of the neural mechanisms of decision-making in social interactions is crucial for understanding the formation and maintenance of personal relationships of positive and negative valence (Güroğlu, van den Bos, & Crone, 2009).

There is substantial amount of research on neural processes underpinning interactions with unfamiliar others (for a review, see Rilling & Sanfey, 2011), yet few neuroscientific studies have investigated social interactions involving familiar others, that is, others from real-life relationships. There are several neuroimaging studies in which decisions concerning friends were compared with those concerning unfamiliar others (Fareri, Chang, & Delgado, 2015; Fareri & Delgado, 2014). Especially, little is known about the underlying neural processes of social decisions involving *disliked peers*, even though it is as crucial to understand a disliked other’s intentions and to act on them in social interactions as it is to understand friends. The majority of prior studies examining decision-making processes with different types of interaction partners have employed experimental manipulations to create positive or negative impressions about unfamiliar others (Bault, Pelloux, Fahrenfort, Ridderinkhof, & van Winden, 2015; Fahrenfort, Pelloux, Stallen, & Ridderinkhof, 2012; Fareri, Chang, & Delgado, 2012; van den Bos, van Dijk, & Crone, 2012). As informative as studies using manipulations of whether one feels positive or negative valence toward others are, the interactions with such unfamiliar others might not be as personally relevant for individuals as are interactions with others from real-life relationships and are hence ecologically less valid. The goal of the current study was thus to investigate how real-life relationships with friends and disliked peers modulate prosocial behavior and the underlying neural processes during these social decisions.

## Social decision-making and its neural correlates

Social interactions involve exchanges with others who might have different intentions and perspectives. People have to rely on inferences about others’ intentions and perspectives in order to guide decision-making in these social interactions (V. K. Lee & Harris, 2013). Using economic allocation paradigms, researchers have shown that in interactions with unfamiliar others, individuals show concern not only for their own outcomes but also for those of their interaction partner (Camerer, 2003; Will & Güroğlu, 2016). Thinking about other people’s mental states, needs, and intentions (i.e., mentalizing) and taking their perspectives into account contribute to the ability to feel concern for others (Batson, Eklund, Chermok, Hoyt, & Ortiz, 2007). These abilities have been consistently linked to activity in a brain network comprising the medial prefrontal cortex (mPFC), temporoparietal junction (TPJ), and superior temporal sulcus (STS; Blakemore, 2008; Frith & Frith, 2012).

Showing concern for others can be expressed by prosocial decisions that (also) benefit others. Prosocial decisions involve self-regulation in the form of controlling selfish impulses (Blake, Piovesan, Montinari, Warneken, & Gino, 2015; Eisenberg, Fabes, Guthrie, & Reiser, 2000; Spitzer, Fischbacher, Herrnberger, Gron, & Fehr, 2007; Steinbeis, Bernhardt, & Singer, 2012), mentalizing skills to shift the attention from the self to the needs and goals of others (e.g., Telzer, Masten, Berkman, Lieberman, & Fuligni, 2011), and possibly a sense of reward (Declerck, Boone, & Emonds, 2013; Zaki & Mitchell, 2011). This is supported by evidence showing involvement of ventrolateral, dorsolateral, and dorsomedial prefrontal cortex (vlPFC, dlPFC, and dmPFC), the TPJ, and the striatum in making prosocial decisions. These are brain regions often implicated in higher order cognitive functions such as self-regulation (vlPFC and dlPFC; Coutlee & Huettel, 2012; Sanfey, Rilling, Aronson, Nystrom, & Cohen, 2003), social cognition (dmPFC and TPJ; Telzer et al., 2011; Waytz, Zaki, & Mitchell, 2012), and reward processing (striatum; Bhanji & Delgado, 2014; Fehr & Camerer, 2007; Izuma, Saito, & Sadato, 2008; Telzer, Fuligni, Lieberman, & Galván, 2013; Telzer et al., 2011).

Activity in the brain regions typically involved in social cognition, such as the mPFC, the STS, and the TPJ, have been shown to be modulated by the relationship valence with the interaction partner during social interactions. For example, TPJ and STS activation has been shown to increase during social interactions with familiar peers compared with unfamiliar others (Güroğlu et al., 2008). Moreover, the social tie with an unfamiliar peer, which develops during interactive social decisions, is shown to modulate activity in the posterior STS (pSTS) and TPJ (Bault et al., 2015; Fahrenfort et al., 2012). That is, lower levels of activation in pSTS and TPJ have been found in interactions with liked others (Bault et al., 2015), and higher levels of pSTS activation have been found when gaining money at the expense of others, but only after a social tie has been established (Fahrenfort et al., 2012). Along these lines, activation in pSTS has been suggested to be involved in keeping track of one’s own and others’ social decisions and their effect on the social interaction (Hampton, Bossaerts, & O’Doherty, 2008). Finally, mPFC activation has often been linked to the integration of (social) information in goal-directed behavior (Amodio & Frith, 2006; Bault, Joffily, Rustichini, & Coricelli, 2011; Bault et al., 2015; Euston, Gruber, & McNaughton, 2012; Sul, Güroğlu, Güroğlu, & Chang, 2017), and its activation is shown to be heightened during decisions involving friends (Braams et al., 2014; Fareri & Delgado, 2014; Güroğlu et al., 2008).

Interaction partners modulate brain activation not only during decision-making in social interactions but also during processing outcomes for others. Processing outcomes are often examined based on distribution of resources or on winning or losing resources (i.e., typically money). Both monetary gains for the self (Fareri et al., 2012; Fareri & Delgado, 2014) and others, such as charities (Kuss et al., 2013; Moll et al., 2006) and family members (Telzer et al., 2011), lead to enhanced activity in the striatum. Social rewards, such as having a good reputation or receiving approval, also lead to enhanced activity in the striatum (Bhanji & Delgado, 2014; Izuma et al., 2008; Jones et al., 2014). Interestingly, heightened striatum activity is associated with observing both monetary gains for friends (Braams et al., 2014; Varnum, Shi, Chen, Qiu, & Han, 2014) and losses for *unfamiliar* disliked others (Braams et al., 2014). In short, these prior studies show that whether one feels positive or negative valence toward interaction partners modulates activity in a set of brain regions implicated in socio-cognitive and emotional processing. The current study is different from these existing studies in that we examine (a) interactions with friends *and* familiar (i.e., real life) disliked peers and (b) active decision-making (i.e., prosocial and selfish decisions) instead of observing monetary outcomes without being able to actually influence them.

Not only does the social context modulates social behavior and its underlying neural processes, but individual differences in prosociality may also affect neural processes during social interactions. In particular, individual differences in social norms and preferences shape neural processes underlying social decision-making in varying social contexts. For example, studies on social exchanges with unfamiliar peers show that individual differences in prosocial behavior related to TPJ involvement when participants made donating decisions while being evaluated by peers (van Hoorn, van Dijk, Güroğlu, & Crone, 2016) and that enhanced activity in the dorsal anterior cingulate cortex (dACC), anterior insula, and dlPFC underlie violations of personal norms in prosocial and selfish decision-making (Güroğlu, van den Bos, Rombouts, & Crone, 2010; Haruno, Kimura, & Frith, 2014; van den Bos, van Dijk, Westenberg, Rombouts, & Crone, 2009).

## The current study

Based on evidence showing that interaction partners modulate prosocial behavior such that individuals are more prosocial toward close others and people they like than more distant and disliked others (Güroğlu et al., 2014a), in this study we investigated whether and how activation of brain regions involved in higher order cognitive functions, mentalizing, and emotion processing are modulated by interaction partners and individual differences in prosociality during social decision-making. In this study, participants actively made prosocial or selfish decisions involving familiar peers who were their actual classmates in real life. By doing so, we aimed to investigate the role of personal relationships of positive (i.e., friends) and negative valence (i.e., disliked peers) in social decisions and the underlying neural circuitry.

To identify existing positive and negative relationships, we used a widely established sociometric nomination method (Cillessen & Bukowski, 2000). Using this method, we were able to identify friends and disliked peers in a group of college students. During the scanning session, participants distributed coins between themselves and another player by choosing one of two preset distributions of coins, where one option always involved a prosocial and the other a selfish distribution of coins. Prosocial distributions benefited the interaction partner irrespective of the costs attached to the decision (Eisenberg et al., 2006), and selfish distributions maximized the outcome of the participant or resulted in the smallest number of coins for the interaction partner possible.

We expected participants to make more prosocial decisions toward their friends than toward disliked peers (Güroğlu, et al., 2014a), and that individual differences in prosociality would relate to brain regions that are sensitive to personal social norms and preferences such as the dACC/SMA, the dlPFC, and TPJ (Güroğlu et al., 2010; Haruno et al., 2014; van den Bos et al., 2009; van Hoorn et al., 2016). We further expected interaction partners to modulate brain activation during decision-making in brain regions involved in social cognition (e.g., self and other preferences and anticipating on outcomes of social decisions), such as the TPJ and STS, the mPFC, and striatum. Specifically, we expected increased mPFC and striatum activity during decisions for friends since these regions have been consistently found to be involved in information processing during interactions with friends (Braams et al., 2014; Fareri & Delgado, 2014; Güroğlu et al., 2008).

## Method

### Participants

Participants were recruited from vocational universities that offer a 4-year bachelor’s degree and have a fixed classroom structure. Students from 24 classrooms in five vocational universities (total *N* = 380) filled out a sociometric questionnaire and an MRI screening checklist. Only right-handed students without a history of psychiatric and neurological impairments were further contacted. Individuals were eligible to participate in the study if they nominated at least two classmates as friends and two classmates as disliked peers. One participant was excluded due to excessive movement in the MRI scanner (>3 mm). The remaining sample consisted of 27 participants (*M*_age_ = 21.25 years, *SD* = 2.93, 15 males).

### Procedure

Before scanning, participants gave their written informed consent to participate, were familiarized with the scanner environment using a mock scanner, and practiced the fMRI task. They received €30 plus their earnings from the fMRI task.

### Sociometric nominations

The sociometric questionnaire was administered in the classroom (class size ranged between 17 and 33 students, *M* = 25.08, *SD* = 4.61). All students in the class were asked to (a) rate how much they like each of their classmates on a 5-point scale, ranging from 1 (*not at all*) to 5 (*very much*), and (b) make five nominations among their classmates for the questions “Who are your friends?” and “Who do you like the least?” These ratings and nominations were used to determine three types of classmates: (a) *Friends* were nominated as friends and received a rating of 4 or 5, (b) *disliked peers* were nominated as least liked classmates and/or received a rating of 1 or 2, and (c) *neutral peers* were classmates receiving a rating of 3. These nominations were used to form the peer groups that the participant played the coin-distribution game with (described below). For each participant, we aimed to have two or three friends and two or three disliked peers. The majority (67.9%) of the friendships that we identified were based on mutual friendship nominations; in total, 79.5% of the nominated friends reported to like the participant very much, and for the remaining 20.5% of the friendships mutuality could not be determined due to missing sociometric data. Relationships based on dislike were more heterogeneous: only 13% of these relationships were based on mutual dislike nominations. In total, 23.2% of the disliked peers reported to dislike the participant or reported *not* to prefer to collaborate with the participant, 42% of the relationships were based on unilateral dislike, and for the remaining 34.8% of the relationships, mutuality could not be determined due to missing sociometric data.

### FMRI task description

#### Peer groups

Participants were told that they would play a coin-distribution game with other peers who were distributed into four groups. They were told that three of these four groups involve randomly chosen peers from their classroom (i.e., classmates) and that the fourth group consists of unfamiliar peers of same age who are also participants of the study. In reality, the group compositions were not random and were based on the sociometric questionnaire. Unique groups of peers were constructed for each participant based on their individual sociometric nominations and ratings. We aimed to have three peer names in the friend and the disliked peer groups; whenever this was not possible, participants were presented with two friend names (11.1%) and two disliked peer names (44.4%). Overall, we presented two groups with two peer names and two groups with three peer names to keep a balanced distribution across the four groups of peers.

Participants were told that on each trial they would see the group they would be distributing the coins with, and the names of the peers in that group, but that they would not exactly know with whom from that peer group they played on each trial. There were three reasons for this manner of presenting the players: (a) to prevent that participants could use strategies of how to distribute coins to different players, (b) to correct for slightly different personal relationships the participant might have with specific players within a group, and (c) to make the task more engaging such that participants did not have to make the same decision for the same player repeatedly. Participants were also told that the computer would keep track of exactly whom they are making a decision for.

**Fig. 1:**
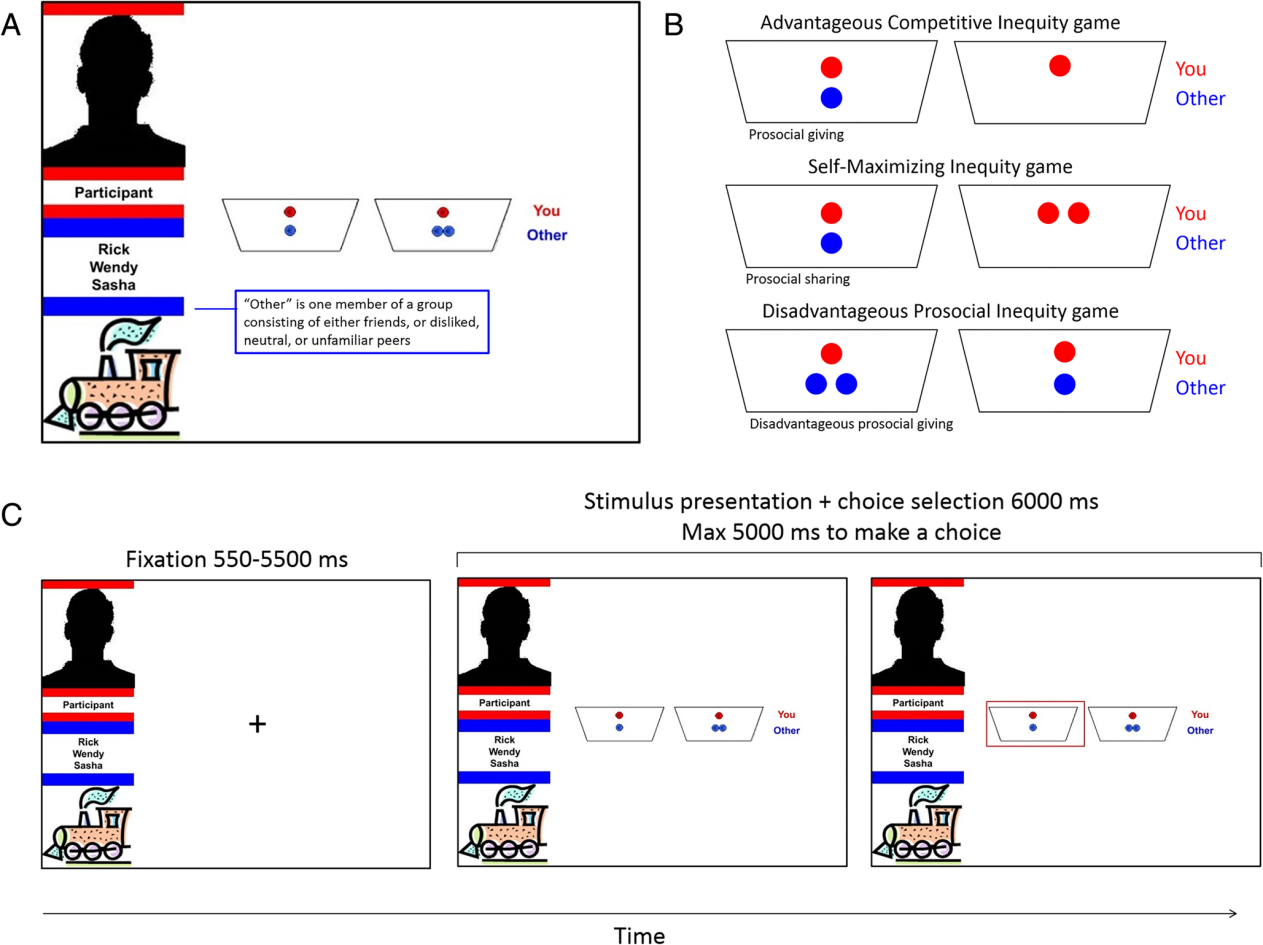
**a** Group member names were displayed on-screen. These three group members always belonged to the same peer category (i.e., friend, disliked peer, neutral peer, or unfamiliar peer). The interaction partner was one of these three group members. **b** There were three different preset coin distributions, always with a prosocial and a selfish option, depicted here on the left and right, respectively. **c** Example of a trial of the fMRI task. After a fixation cross, participants were presented with a screen showing the stimulus and with whom they were playing that trial. At stimulus onset, they could choose between the two options presented on the screen by pressing the corresponding button. A trial ended with selected choice indicated on the screen. (Color figure online)

In order to present the four groups of friends, disliked peers, neutral peers, and unfamiliar peers in a neutral manner to the participants, the groups were randomly assigned to one of the four vehicle symbols named train, bike, car, and boat (see Fig. 1a). The names of the group members were presented to the participants at the start of the scanning session (before scanning started). Participants were told that they were not required to memorize these names and that the names would be presented on the screen during each trial of the task.

At the end of the experiment, a free-recall test was administered to see whether the participants could produce the names of the group members for each of the four groups of interaction partners. They were also asked about their attitude toward each group by writing down what they thought of the members of each group. This was done to check whether the manipulation of groups representing different kinds of relationships was successful and to assess whether the participants paid attention to the task. Results of the manipulation checks are reported in the Results section.

#### Coin distributions

Participants played three modified dictator games (Fehr, Bernhard, & Rockenbach, 2008; Güroğlu, Will, & Crone, 2014b), in which they distributed coins between themselves and another player. In each of the games, participants were asked to choose one of two predetermined distributions of coins. Each game had one prosocial option and one selfish option: (a) In the advantageous competitive inequity (ACI) game, participants could choose to keep one coin for themselves and give nothing to the other player (self/other: 1/0, selfish option) or give one coin to the other player, resulting in an equal distribution (1/1, prosocial option); (b) in the self-maximizing inequity (SMI) game, participants could choose to keep two coins for themselves (2/0, selfish option) or share the two coins with the other person, resulting in an equal distribution (1/1, prosocial option); and (c) in the disadvantageous prosocial inequity (DPI) game, participants could equally divide two coins between themselves and the other player (1/1, selfish option) or give an additional coin to the other player (1/2, prosocial option). Prosocial choices in the three games were coded as 1, and selfish choices were coded as 0. We used these different types of prosocial choices (i.e., prosocial giving in the ACI game, prosocial sharing in the SMI game, and disadvantageous prosocial giving in the DPI game) to keep the participants engaged in the task (see Fig. 1b). Percentage of prosocial choices per interaction partner was calculated across games. It was explained that the computer kept track of the coin distributions and calculated everyone’s earnings, which would be paid out at the end of all the trials. During the instructions, it was also emphasized that decisions had consequences for the participants as well as for the interaction partners. However, it was not explicitly specified how this would exactly be implemented; none of the participants had questions about this implementation. In reality, all participants got feedback at the end of the task that they had earned €2.

#### Task duration

The task consisted of 96 trials presented in a randomized order, in which participants engaged in 24 interactions with members of each group across a set of three allocation games. Each trial started with a jittered fixation cross (*M* = 1,512.5 ms, min = 550 ms, max = 5,500 ms; optimized with Opt-Seq2, surfer.nmr.mgh.harvard.edu/optseq/) (Dale, 1999). This was followed by a screen with the group symbol and its members’ names and the set of distributions they could choose from (see Fig. 1c). Participants had 5,000 ms to respond by a button press with their right index finger for the distribution on the left and with their right middle finger for the distribution on the right. The response of the participants was presented on the screen until 6,000 ms. If they failed to respond within 5,000 ms, a screen showing “Too late!” was presented for 1,000 ms. The location of the equity option was counterbalanced across trials.

### MRI data acquisition

MRI scans were acquired using a 3T Philips Achieva MRI scanner. The scanning procedure included a localizer scan, and T2* weighted gradient echo planar images (EPI) (TR = 2.2 s, TE = 30 ms, descending and sequential acquisition, 38 slices of 2.75 mm, field of view [FOV] = 220 × 220 × 114.7 mm) were obtained during two functional runs. Each run consisted of 170 volumes and lasted approximately 6 minutes.

### FMRI data analysis

Image preprocessing and analyses were conducted using SPM8 software (http://www.fil.ion.ucl.ac.uk/spm/). The preprocessing steps of the functional images included realignment, slice-time correction (middle slice as reference), spatial normalization to EPI templates, and smoothing with a Gaussian filter of 8 mm full-width at half maximum. Regressors were modeled as zero-duration events (stick functions) time locked to the stimulus onset and convolved with a canonical hemodynamic response function; stimulus onset was the moment participants were presented with the two distributions to choose from. Trials on which the participant failed to respond were modeled separately as covariate of no interest and were excluded from further analyses. The modeled events (players; i.e., friends, and disliked, neutral, and unfamiliar peers, and type of response; i.e., prosocial or selfish, per player) were used as regressors in a general linear model (GLM), along with a basic set of cosine functions that high-pass filtered the data (cutoff 120 seconds) and a covariate for session effects. Autocorrelations were estimated using an AR(1) model. The least-square parameter estimates of height of the best fitting canonical HRF for each condition were used in the contrasts. No events for the button press were included in the GLM. For visualization purposes, mean beta estimates were extracted from whole-brain clusters using the MarsBaR toolbox (Brett, Anton, Valabregue, & Poline, 2002). Activity was averaged across the clusters derived from our whole-brain analyses. All results are reported in Montreal Neurological Institute 305 stereotactic space.

We examined the neural underpinnings of decision-making for friends and disliked peers by comparing (a) the two most “extreme” relationships (i.e., friendships and relationships based on dislike) and by comparing (b) decisions involving friends and disliked peers with decisions involving peers with whom participants had no affective relationship, that is, the unfamiliar peers. For these comparisons we used the unfamiliar peers instead of the neutral peers because none of the participants was affiliated with unfamiliar peer in any way, making these relationships more homogeneous across the participants. We report the contrasts with neutral peers in the Supplementary Material. Also see the Supplementary Materials for whole-brain contrasts of decision-making for different types of peers collapsed across behavior (i.e., the general Friend > Disliked Peer, Friend > Unfamiliar Peer, Disliked Peer > Friend, and Disliked Peer > Unfamiliar Peer contrasts).

To examine how prosocial tendencies to different types of interaction partners relate to the underlying neural process, we examined brain and behavior links with (a) percentage of prosocial choices for friends minus disliked peers as a regressor in the Friend > Disliked Peer whole-brain *t* contrast, (b) percentage prosocial choices for friends minus unfamiliar peers as a regressor in the Friend > Unfamiliar Peer whole-brain *t* contrast, and (c) percentage of prosocial choices for disliked peers minus unfamiliar peers as a regressor in the Disliked Peer > Unfamiliar Peer whole-brain *t* contrast.

Next, we conducted analyses in which we broke down the Friend > Disliked Peer, Friend > Unfamiliar Peer, Disliked Peer > Friend, and Disliked Peer > Unfamiliar Peer contrasts by behavior to examine the neural activation underlying prosocial and selfish choices in interactions with friends and disliked others. We did this by contrasting prosocial choices for friends with those for disliked peers (Friend Prosocial > Disliked Peer Prosocial) and unfamiliar peers (Friend Prosocial > Unfamiliar Peer Prosocial), and by contrasting prosocial choices for disliked peers with those for friends (Disliked Peer Prosocial > Friend Prosocial) and unfamiliar peers (Disliked Peer Prosocial > Unfamiliar Peer Prosocial). Similarly, we examined the contrasts for selfish choices, that is, Friend Selfish > Disliked Peer Selfish, Disliked Peer Selfish > Friend Selfish, Friend Selfish > Unfamiliar Peer Selfish, and Disliked Peer Selfish > Unfamiliar Peer Selfish. In all these contrasts, we controlled for the percentage of the behavior of interest. For example, we controlled for the percentage of prosocial choices in the Friend Prosocial > Disliked Peer Prosocial contrast by first subtracting the percentage of prosocial choices for disliked peers from the percentage of prosocial choices for friends for each participant, and then by including these values as a covariate in the whole-brain contrasts. We did the same thing for social decision-making with disliked peers.

**Fig. 2:**
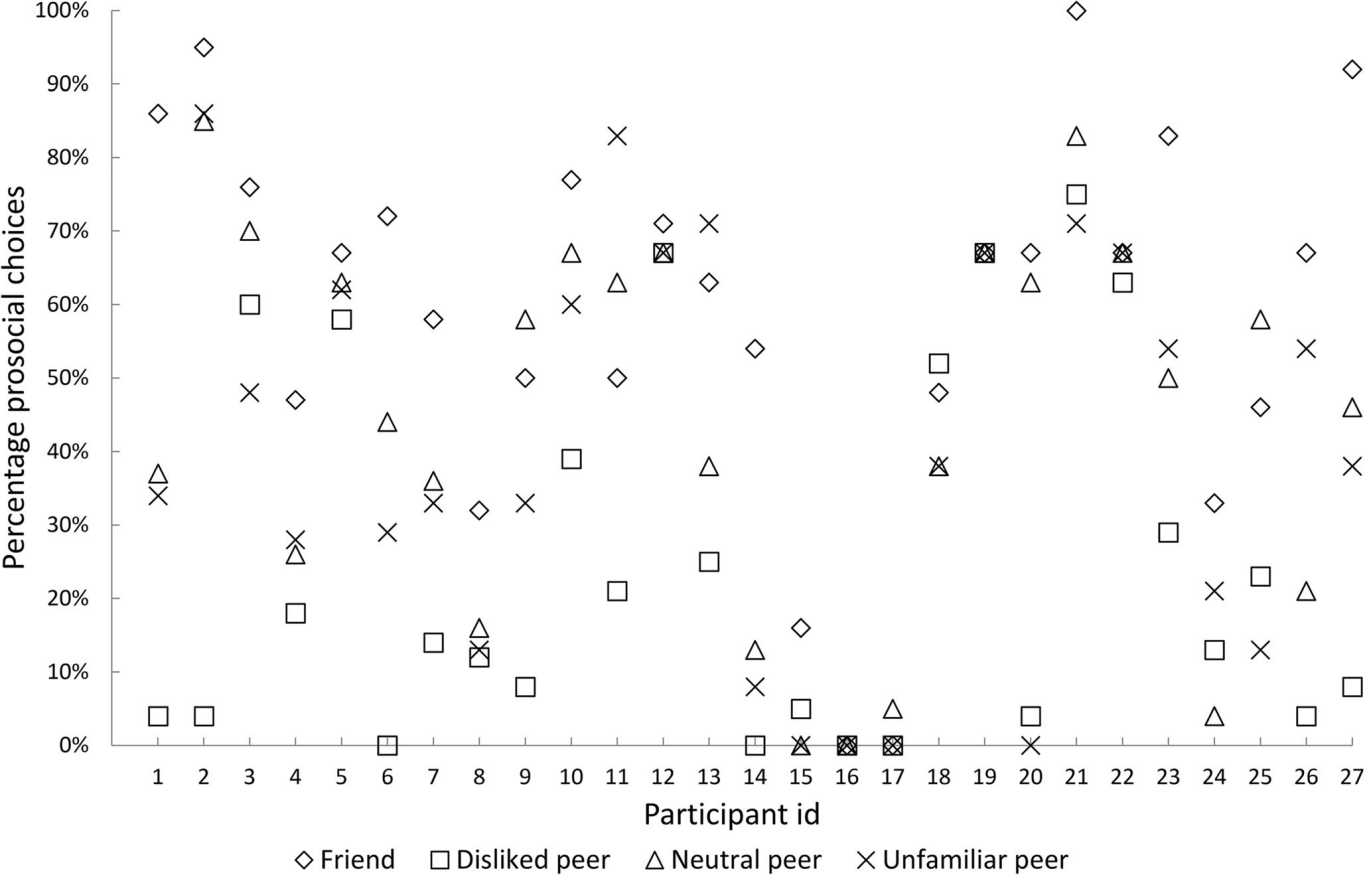
Percentage of prosocial choices separately for friends, disliked peers, neutral peers, and unfamiliar peers for each of the 27 participants

Importantly, these analyses are considered preliminary because (a) the sample size in the analyses contrasting prosocial and selfish decisions might differ from the complete sample size of 27 participants due to participants who did not make the specific decision of interest and could thus not be included in a specific contrast, and (b) we did not exclude participants from the analyses based on a minimum number of responses in a specific contrast. The latter decision was made because (a) we wanted to make use of our full data set in our relatively small sample, and (b) participants with few trials in a specific contrast are also those who are consistent in their behavior toward different types of peers (e.g., by being consistently selfish toward disliked peers or prosocial toward friends) and thus of interest for our research questions. Figure 2 shows for each participant the percentage of prosocial choices made for friends, disliked peers, neutral peers, and unfamiliar peers. See also Table S1 Supplementary Materials for an overview of how many participants had more than zero, one, two, three, four, or five trials in the contrasts, discussed in the Results section. To further check the robustness of these results, we also report our results where we reran these analyses with a subset of the sample.

We considered the results significant using family-wise error (FWE) cluster-correction at *p* < .05, with a cluster-forming threshold of *p* < .005 (Woo, Krishnan, & Wager, 2014). We chose a threshold of *p* < .005 to avoid Type II errors (Lieberman & Cunningham, 2009). This correction method has greater sensitivity to weak and diffuse signals and is suitable for relatively small sample sizes (*N* < 50; Cremers, Wager, & Yarkoni, 2017; Woo et al., 2014).

## Results

### Manipulation check

Correct recall of the names of the interaction partners (“players”) was high (*M*_range_ = 87%–91%; *SD*_range_ = 20%–30%). There were no significant differences in percentage of correct recall of the names in the four groups, *F*(2.23, 55.70) = .16, *p* = .87, Greenhouse–Geisser corrected. Open-ended questions about how participants described the four groups were coded into a 5-point scale, ranging from 1 (*very negative*) to 5 (*very positive*). There were significant differences between attitudes to the familiar peers (i.e., friends, disliked peers, and neutral peers), *F*(2, 46) = 125.66, *p* < .001, η_p_^2^ = .845. Participants evaluated friends (*M* = 4.58, *SE* = .10) more positive than neutral peers (*M* = 3.46, *SE* = .10), which were also evaluated more positive than disliked peers (*M* = 2.13, *SE* = .14), all *p*s < .001. For the unfamiliar peers, 18 participants (66.7%) stated “these persons were unfamiliar”; eight (29.6%) participants described them as neutral (*M* = 3.38, *SD* = .74), and one participant (3.7%) was missing a description. This manipulation check confirmed that participants differentiated between the four groups regarding their relationship with the players in each group.

### Behavioral results

**Fig. 3:**
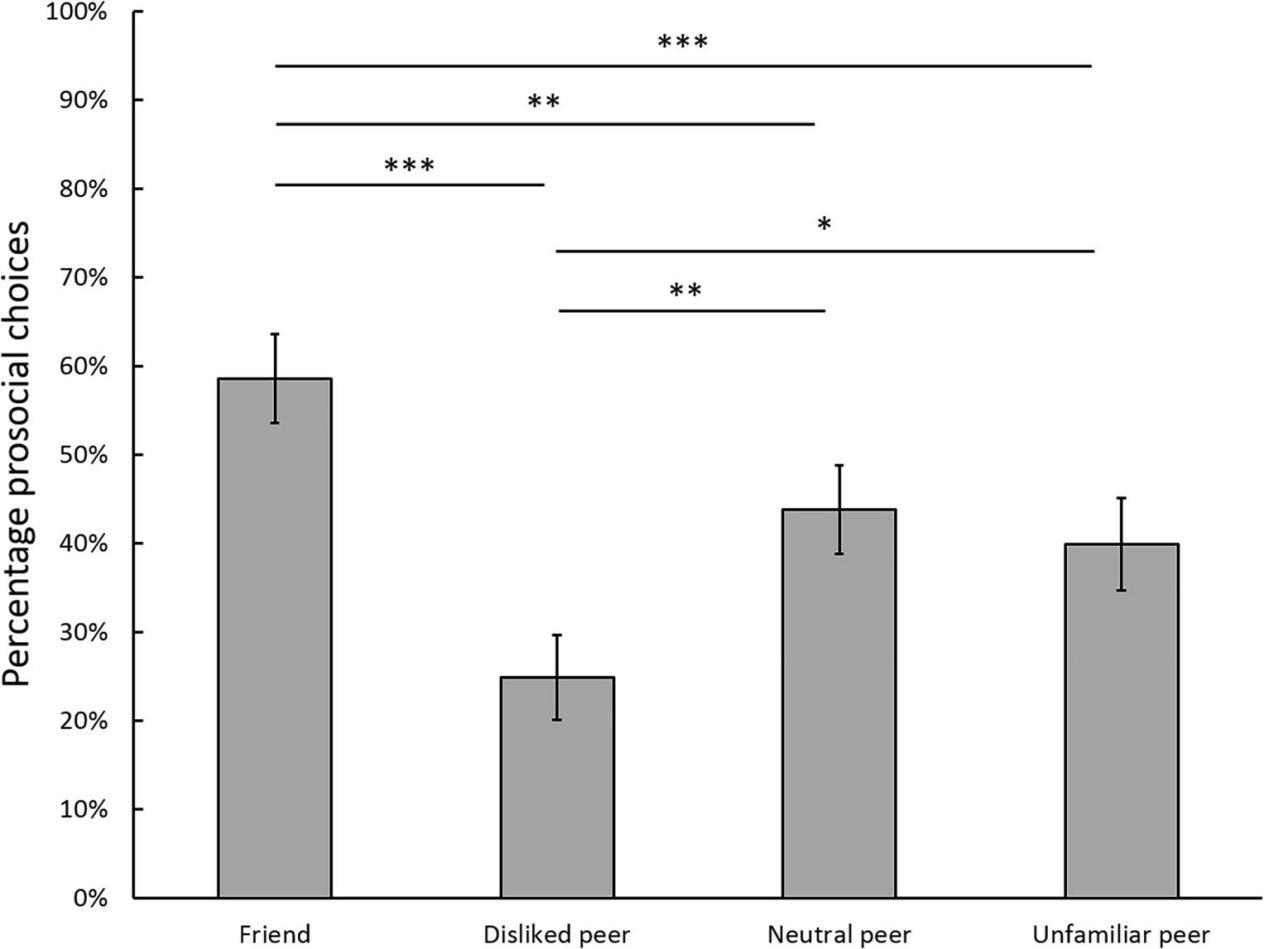
Mean frequency (%) and standard errors (as indicated by the error bars) of prosocial choices per interaction partner. Significant differences are indicated by an asterisk (*). *p < .005, ** *p* < .01, *** *p* < .001

An examination of participants’ individual response patterns in the fMRI task showed that they had strong preferences for prosocial or selfish choices depending on their interaction partner (see Fig. 2 for a detailed overview of frequencies at trial level). To examine whether participants’ prosocial behavior was modulated by the interaction partner, a repeated-measures ANOVA was conducted, with within-subject factor player (four levels: friend, disliked peer, neutral peer, and unfamiliar peer), and the percentage of prosocial choices as the dependent variable. There was a significant main effect of player, *F*(3, 78) = 20.487, *p* < .001, η_p_^2^= .441. Post hoc tests for this main effect showed that participants made significantly more prosocial choices when they were playing for friends (*M* = 59%) than for disliked peers (*M* = 25%, *p* < .001), neutral peers (*M* = 44%, *p* < .01) and unfamiliar peers (*M* = 40%, *p* < .001), and when playing for neutral peers and unfamiliar peers than for disliked peers, *p* < .01 and *p* < .05, respectively. Prosocial behavior toward unfamiliar and neutral peers did not differ significantly from one another, *p* = 1. These results demonstrate that participants were more prosocial toward friends and less prosocial toward disliked peers than toward other peers (see Fig. 3). There were no significant differences in response time for decisions for the players, *F*(3, 78) = 2.548, *p* = .06.

### Neuroimaging results

#### Links between individual differences in prosocial behavior and neural processes

**Fig. 4:**
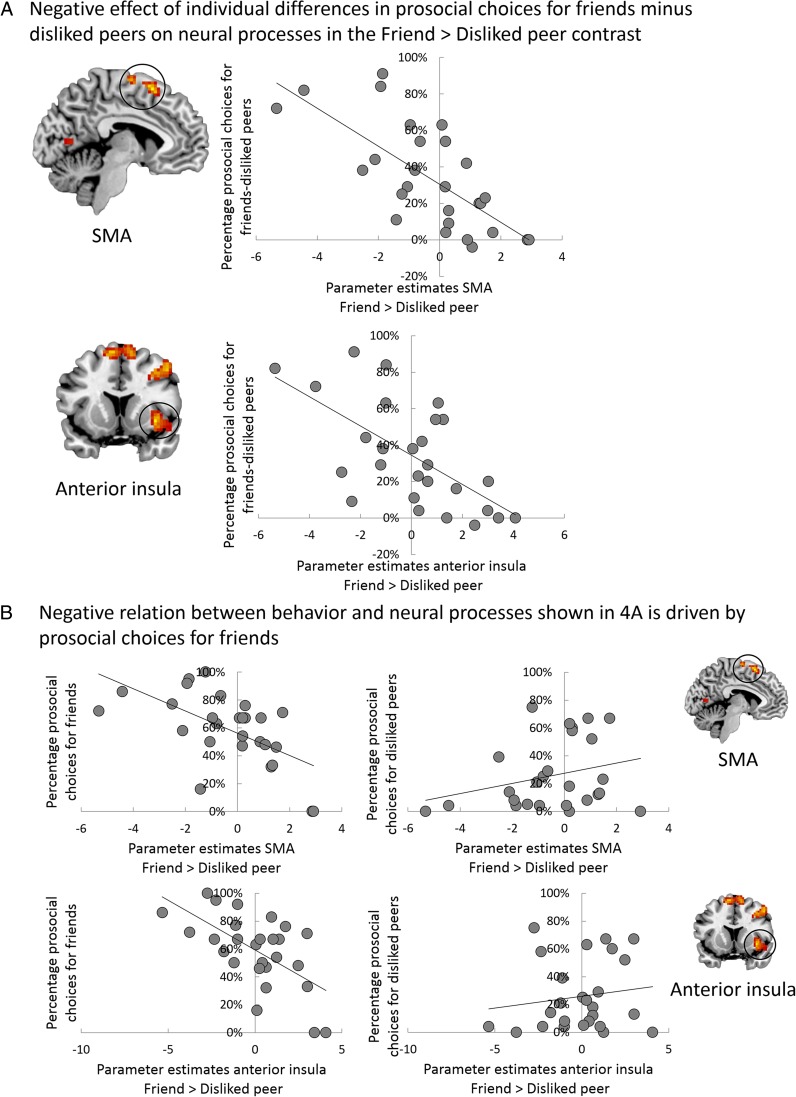
**a** Percentage of prosocial choices for friends minus disliked peers as a negative regressor in the whole-brain contrast Friend > Disliked Peer resulted in right anterior insula (36, 12, −6) and supplementary motor area (SMA) activation (−6, 15, 60). **b** Parameter estimates of the beta values of SMA and anterior insula from this contrast are plotted for percentage of prosocial choices for friends (left panel) and disliked peers (right panel) separately, showing that the negative relation between prosocial choices for friends minus disliked peers with SMA and anterior insula is driven by prosocial choices for friends. (Color figure online)

In order to investigate brain and behavior links during interactions with friends and disliked peers separately, we included the difference scores of the percentage of prosocial choices for friends and disliked peers as a regressor in the Friend > Disliked Peer *t* contrast (see Table 1). This revealed a negative correlation between the number of prosocial decisions for friends minus disliked peers and activity in the supplementary motor area (SMA) and right anterior insula (see Fig. 4a). To inspect whether this negative relation was driven by individual differences in prosocial choices for friends or disliked peers, we plotted the mean parameter estimates of the beta values against the percentage of prosocial choices for friends and disliked peers separately (see Fig. 4b). These plots show that the negative relation between percentage of prosocial choices for friends minus disliked peers and SMA and anterior insula activity is driven by prosocial interactions with friends: correlation coefficients of the relation between the parameter estimates of the SMA and anterior insula of the Friend > Disliked Peer contrast and (a) the percentage of prosocial choices for friends are −.60 and −.62, respectively, and (b) the percentage of prosocial choices for disliked peers are .27 and .15, respectively. These analyses did not yield any positive correlations.

**Table 1:** Regions of neural activation of correlations between prosocial choices and whole-brain *t* contrasts

Brain region	L/R	Voxels	*z*	MNI coordinates
				*x y z*
Friend > Disliked peer				
*Mean prosocial choices for friends-disliked peers as negative regressor*				
Supplementary motor area (SMA)	–	511	4.10	−6 15 60
			3.87	15 9 60
			3.86	21 0 66
Anterior insula	R	171	4.05	36 12 −6
			3.40	51 15 −18
			2.86	30 21 12
Middle frontal gyrus	R	208	3.83	48 12 45
			3.58	36 12 45
			3.36	39 −18 39
Calcarine gyrus	R	126	3.67	15 −72 18
			3.45	24 −69 12
			3.34	18 −81 12
Precentral gyrus	L	149	3.48	−45 6 48
			3.38	−66 −27 30
			3.26	−42 −12 42
Lingual gyrus	L	142	3.42	−18 −63 −12
			3.22	−18 −69 12
			3.03	−24 −54 −9

*Note.* MNI = Montreal Neurological Institute. Analyses are conducted using FWE cluster correction at *p* < .05, with a cluster-forming threshold of *p* < .005

Analyses using the difference scores of percentage of prosocial choices for friends minus unfamiliar peers as a regressor in the Friend > Unfamiliar Peer and the difference scores of percentage of prosocial choices for disliked peers and unfamiliar peers as a regressor in the Disliked Peer > Unfamiliar Peer *t* contrasts did not result in any significant positive or negative relations with brain activity at our chosen threshold.

#### Prosocial and selfish choices

Next, we examined neural activation patterns for specific behaviors (i.e., prosocial or selfish) separately for friends and disliked peers. Note that sample sizes for these results diverge from our total sample size of 27 due to participants who never make specific choices (e.g., prosocial choice for disliked peer).

#### Friends

**Fig. 5:**
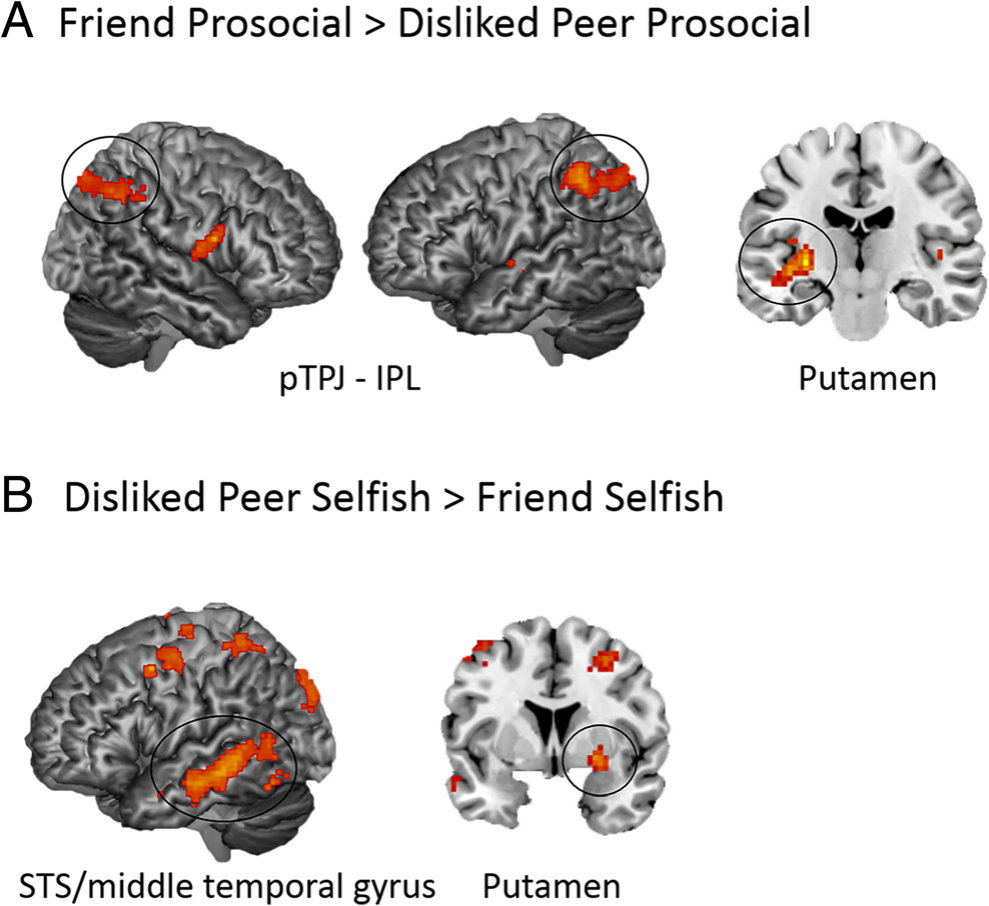
**a** Whole-brain *t* contrasts controlling for the percentage of prosocial choices for Friend Prosocial > Disliked Peer Prosocial, which resulted in bilateral pTPJ–IPL (45, −57, 45; −48, −48, 48) and left putamen activation (−30, −18, 0). **b** Whole-brain *t* contrasts for Disliked Peer Selfish > Friend Selfish, controlling for the percentage of selfish choices, resulted in activation in left STS/middle temporal gyrus (−66, −36, 0) and right putamen (24, 3, −6). pTPJ = posterior temporoparietal junction; IPL= inferior parietal lobule; STS = superior temporal sulcus

We investigated neural activation during interactions with friends separately for prosocial and selfish choices. The Friend Prosocial > Disliked Peer Prosocial contrast (*n* = 23), controlling for the percentage of prosocial choices, resulted in activation in left putamen, and left inferior parietal lobule (IPL) and right IPL extending toward the angular gyrus (see Fig. 5a). These parietal brain regions have been previously labeled as subdivisions of the TPJ, and will be henceforth referred to as posterior TPJ (pTPJ)–IPL (Mars et al., 2012). The Friend Prosocial > Unfamiliar Peer Prosocial (controlling for the percentage of prosocial choices, *n* = 23) yielded activation in a cluster containing the left IPL extending toward the superior parietal lobule (SPL), precuneus, and angular gyrus, and right IPL extending toward the angular gyrus. These regions are henceforth also referred to as pTPJ–IPL. The Friend Selfish > Disliked Peer Selfish and Friend Selfish > Unfamiliar Peer Selfish contrasts did not result in significant clusters of activation at our chosen threshold. See Table 2 for a detailed overview of the results.

**Table 2:** Regions of neural activation for friends and disliked-peer whole-brain *t* contrasts controlled for the percentage of behavior of interest

Brain region	L/R	Voxels	*z*	MNI coordinates
				*x y z*
Prosocial choices				
*Friend > Disliked Peer*				
Putamen	L	160	3.92	−30 −18 0
			3.77	−24 −9 −6
			3.50	−39 −15 −6
pTPJ–IPL	L	297	3.88	−48 −48 48
			3.49	−27 −57 42
			3.26	−36 −60 45
pTPJ–IPL	R	149	3.23	45 −57 45
			3.19	36 −72 51
			3.16	42 −51 39
Inferior frontal gyrus–Rolandic operculum	R	121	3.73	51 6 18
			3.55	48 −6 15
			3.31	36 0 18
*Friend > Unfamiliar Peer*				
pTPJ–IPL	L/R	594	3.86	9 −75 45
			3.55	−33 −69 42
			3.45	−42 −54 42
pTPJ–IPL	R	277	3.84	36 −69 45
			3.67	51 −42 54
			3.59	45 −54 57
Selfish choices				
*Disliked Peer > Friend*				
Middle temporal gyrus–superior temporal sulcus	L	487	4.63	−66 −36 0
			4.46	−66 −30 −6
			4.01	−57 −18 −15
Putamen		142	3.78	24 3 −6
			3.46	30 −3 −24
			3.40	27 −6 −12
Postcentral gyrus–precentral gyrus		2081	4.58	45 −21 48
			4.38	−12 −27 60
			4.30	−48 9 51
Middle temporal gyrus	R	164	4.15	60 −63 0
			3.71	48 −60 6
			3.26	54 −57 12
Occipital gyrus	L	244	3.87	−15 −90 33
			3.26	−9 −78 15
			3.19	−30 −87 24
Lingual gyrus		423	3.79	24 −51 0
			3.70	12 −36 −3
			3.69	21 −60 15

*Note.* MNI = Montreal Neurological Institute; pTPJ = posterior temporoparietal junction; IPL= inferior parietal lobule. Analyses are conducted using FWE cluster-correction at *p* < .05, with a cluster-forming threshold of *p* < .005

#### Disliked peers

We conducted one-sample *t* tests to investigate neural activation for disliked peers during prosocial and selfish choices separately. The Disliked Peer Selfish > Friend Selfish contrast, controlling for percentage of selfish choices (*n* = 26), yielded activation in the left middle temporal gyrus/STS, and right putamen (see Fig. 4b). The Disliked Peer Prosocial > Friend Prosocial, Disliked Peer Prosocial > Unfamiliar Peer Prosocial, and Disliked Peer Selfish > Unfamiliar Peer Selfish contrasts did not result in heightened brain activation. See Table 2 for a detailed overview of the results.

#### Robustness of results

To examine the robustness of these results, we reran these analyses where we excluded participants who only had one trial for a specific contrast. In the Friend Prosocial > Unfamiliar Peer Prosocial contrast, we replicated enhanced activity in bilateral pTPJ–IPL. Enhanced activity in bilateral pTPJ–IPL in the Friend Prosocial > Disliked Peer Prosocial contrast was only replicated at an uncorrected threshold of *p* < .005. We did not replicate the enhanced putamen activity in the Friend Prosocial > Disliked Peer Prosocial contrast. In the Disliked Peer Selfish > Friend Selfish contrast, we replicated the enhanced STS activity, but the enhanced putamen activity in the Disliked Peer Selfish > Friend Selfish contrast was only replicated at an uncorrected threshold of *p* < .005. Importantly, there were no outliers in the activation patterns in the original Friend Prosocial > Disliked Peer Prosocial and Disliked Peer Selfish > Friend Selfish contrasts, suggesting that differences stem from a decrease in statistical power (see Supplementary Materials for more details).

## Discussion

This study examined the role of real-life relationships with peers during prosocial decisions and their neural correlates in young adults. Participants made more prosocial decisions in interactions with their friends and more selfish decisions (i.e., fewer prosocial decisions) in interactions with disliked peers. Our fMRI findings show that making fewer prosocial decisions for friends was associated with greater SMA and right anterior insula activity during interactions with friends versus disliked peers. We further show with preliminary analyses that putamen activity was elevated when participants made prosocial decisions involving friends and selfish decisions involving disliked peers. Prosocial decisions involving friends were also associated with heightened bilateral pTPJ–IPL activation, and selfish decisions involving disliked peers were associated with heightened STS activation.

When investigating individual differences in neural processes underlying prosocial behavior, we found a negative relation between the percentage of prosocial decisions for friends versus disliked peers and activation in SMA and anterior insula during interactions with friends relative to those with disliked peers. In other words, participants who were less prosocial toward their friends had higher activation in SMA and anterior insula during these interactions. In a prior study in which participants distributed coins between themselves and unfamiliar peers in a similar research paradigm, enhanced activity in the dACC and anterior insula was associated with inequity decisions, which could be either selfish or prosocial in nature (Güroğlu, Will, & Crone, 2014b). The current study extends these results by showing that not acting in a prosocial manner toward friends yields similar neural responses as when distributing coins in an unequal manner with unfamiliar peers.

In previous studies examining the neural correlates of social decision-making, the anterior insula and dACC or SMA are often interpreted to be involved in detecting the violation of social norms and in resolving the motivational conflict (e.g., for a meta-analysis, see Feng, Luo, & Krueger, 2015). Likewise, activity in the dACC and anterior insula are also interpreted to be involved in personal norm violations, like when prosocial-oriented individuals act selfishly or self-oriented individuals act prosocially (van den Bos et al., 2009), or when individuals make decisions that are not consistent with the socially accepted responses in particular social contexts (Güroğlu et al., 2010). Hence, a possible mechanism that could be underlying the neural response in our participants is that they evaluate their behavior based on their norms when interacting with friends, that is, making a distribution that benefits the friend (i.e., prosocial decisions). It is important to note that the dACC or SMA and insula are implicated in a broad range of cognitive tasks, including conflict monitoring, error detection, and processing pain (Bonini et al., 2014; Eisenberger & Lieberman, 2004; Yarkoni, Poldrack, Nichols, van Essen, & Wager, 2011); however, such other plausible functions of these regions have been interpreted to be in line with their involvement in social norm violations (Feng et al., 2015; Montague & Lohrenz, 2007). One could pose that there is a general social norm to act in prosocial ways toward friends and that, speculatively, not acting according to this social norm could induce internal conflict.

Interestingly, individual differences in prosocial behavior toward friends relative to unfamiliar peers did not yield increased neural activity in interactions with friends compared with unfamiliar peers. Speculatively, the fact that we did not find similar brain and behavior links that may suggest a role of social norm violations in interactions with friends versus unfamiliar peers as in interactions with friends versus disliked peers may be due to differences in socio-emotional valences of the relationships with disliked and unfamiliar peers. Tentatively, results obtained from contrasts in which interactions with friends are compared with those with disliked peers may have a higher socio-emotional valence because one’s behavior in these interactions may affect the relationship, whereas behavior in interactions with unfamiliar peers may not change the relationship because there is no prospect of future social interactions. Furthermore, one might also hold social norms such that one should be nice (i.e., prosocial in this context) to unfamiliar others, which is similar to expectancies for friends. In this respect, it is possible that disliked peers are more distinct from friends than unfamiliar peers are compared with friends. These hypotheses should be tested in future studies.

In the whole-brain contrasts comparing prosocial decisions for friends with prosocial decisions for disliked peers, we found that prosocial interactions with friends involved higher activation of a posterior TPJ region extending towards the IPL (pTPJ–IPL), a subdivision of the TPJ previously found to be connected to the lateral prefrontal cortex (Mars et al., 2012). The pTPJ–IPL region has been shown to be involved in mentalizing processes, such as understanding intentionality and others’ perspectives (Güroğlu, van den Bos, van Dijk, Rombouts, & Crone, 2011; Saxe, 2006; van den Bos, van Dijk, Westenberg, Rombouts, & Crone, 2011; Young, Dodell-Feder, & Saxe, 2010), but also with other cognitive tasks, such as attentional processing (Vossel, Geng, & Fink, 2014), adjusting to a new or changed context (Geng & Vossel, 2013), and memory processes (Anticevic, Repovs, Shulman, & Barch, 2010; Corbetta, Kincade, & Shulman, 2002; for a comprehensive review, see Cabeza, Ciaramelli, & Moscovitch, 2012). Interestingly, it has been argued that the TPJ is involved in integrating distinct streams of attentional and memory processes, which together contribute to processing social contexts (Carter & Huettel, 2013). Involvement of the pTPJ–IPL during prosocial decisions involving friends is consistent with prior studies showing its important role in social interactions (Carter, Bowling, Reeck, & Huettel, 2012; Halko, Hlushchuk, Hari, & Schürmann, 2009) and in prosocial decision-making (van Hoorn et al., 2016). A recent study also shows its involvement in the regulation of social behavior, such that the pTPJ is suggested to facilitate prosocial behavior toward close others but not for distant others (Strombach et al., 2015). Given that pTPJ–IPL activation was enhanced for prosocial decisions for friends when compared to both disliked and unfamiliar peers, our results indicate that the pTPJ is recruited to a greater extent during prosocial interactions with liked and close others compared to distant others such as disliked or unfamiliar peers. Considering the resting-state connectivity of this region with the prefrontal cortex as previously reported by Mars et al. (2012), future research should investigate the connectivity patterns to better understand how this region might support social decision-making.

In the whole-brain contrasts comparing selfish decisions for disliked peers with selfish decisions for friends, we found involvement of the STS during selfish interactions with disliked peers. The STS is involved in social information processing, such as in processing eye contact (Pelphrey, Viola, & McCarthy, 2004), attributing intentions to inanimate objects (S. M. Lee, Gao, & McCarthy, 2014), and understanding and sharing emotions (Paulus, Müller-Pinzler, Jansen, Gazzola, & Krach, 2014; Peelen, Atkinson, & Vuilleumier, 2010; Zaki, Weber, & Ochsner, 2012). Furthermore, the STS is involved in tracking whether expectations about a social response are matched (Hampton et al., 2008). These findings suggest that the STS is involved in mentalizing processes, which might be important for recognizing the type of social setting or dynamic in social settings. Our results are in line with prior studies showing that during social decisions STS activity is modulated by the social relationship with the interaction partner (Bault et al., 2015), and that STS activation is enhanced when gaining money at the expense of others (Fahrenfort et al., 2012). The role of the STS in social interactions with negative valence should be further investigated in future studies to test these interpretations.

The putamen was activated both during prosocial decisions for friends and selfish decisions for disliked peers. Prior studies have also implicated putamen activation in being positively evaluated by peers (Gunther Moor, van Leijenhorst, Rombouts, Crone, & van der Molen, 2010). Similarly, enhanced putamen activation during prosocial decisions has been suggested to be related to predicting and anticipating outcomes of social interactions with peers (Delgado, Frank, & Phelps, 2005). Interestingly, the current study showed that putamen activation was also greater during selfish decisions in interactions with disliked peers than in interactions with friends. In these interactions, the participant chose to decrease the outcomes for the disliked peers. Consistent with this finding, Takahashi et al. (2009) found that activation in the putamen was heightened when envied peers experienced misfortune. Corroborating prior findings, the putamen might be involved in the anticipation of expected pattern of behaviors in social interactions. It would be interesting to further investigate how this might fit with putamen’s role within the striatum in social learning for example in relation to prediction errors.

### Strengths, limitations and conclusions

The current study provides a valuable starting point for future research where ecological validity should be further increased by, for example, having liked and disliked peers present. An advantage of the current research paradigm is that we used sociometric nominations in a closed peer group of college students to identify different types of peer relationships. The current study design enabled us to examine the underlying processes of social decision-making in the real world in an ecologically valid manner. This provides potential insights in how existing relationships are maintained (Güroğlu et al., 2009).

During the task, participants were explicitly instructed to remember that their decisions in the task would not only have monetary consequences for themselves but also for their interaction partners on each trial. Considering that the implementation of the payments for their interaction partners was not explicitly specified, it is plausible that (some) participants might have seen their decisions to be hypothetical. Nevertheless, the behavioral results we present here suggest that participants have taken the task seriously and differentiate between different groups of players as we have expected.

Our behavioral findings showed that the percentage of prosocial decisions differed significantly across the interaction partners, which made it difficult to dissociate effects of behavior and interaction partners. This is in line with prior findings that show that friendships typically involve more prosocial behavior than interactions with disliked others (Newcomb & Bagwell, 1995). Here, we aimed to control for these behavioral differences by including the percentage of prosocial behavior as a covariate in our analyses. However, one might raise the question whether it is favorable to dissociate the percentage of prosocial behavior and the relationship with interaction partners, because the combination of factors might give better insights in the underlying processes involved than the two factors separately.

It should also be noted that our sample size was relatively small (*N* = 27) for analyses of interindividual differences. Therefore, our results linking individual differences in the percentage of prosocial decisions should be interpreted with caution and replicated in future studies. Relatedly, in our analyses we did not exclude participants based on a minimum number of responses in a specific condition. By doing so, we were able to use all the data of our relatively small sample, and we were not forced to create groups of participants with a specific type of social motivation (i.e., generally prosocial or selfish). In our study, participants were generally consistent in their behavior within a certain condition, which indicates that they did not make random choices in the fMRI task. Although this type of behavior is desired, because it reflects stable individual preferences (Güroğlu, Will, & Crone, 2014b), it resulted in imbalanced whole-brain contrasts for some of our analyses. We did not replicate all our fMRI findings obtained from imbalanced whole-brain contrasts when we excluded participants with only one trial for these contrasts. This could be due to a power issue since our findings were not driven by outliers (see Fig. S1in the Supplementary Materials). Nonetheless, the results from the analyses comparing prosocial and selfish decisions for friends and disliked peers should be interpreted with caution and replicated in future studies.

The current study was the first to use an ecologically valid experimental design to investigate neural correlates of prosocial and selfish decisions in interactions with different types of familiar peers, that is, friends and disliked peers. We demonstrate that the personal valence of the relationship with the interaction partner modulates behavior and neural activity in several brain regions typically involved in social cognition. These findings set the stage for future studies to further investigate how real-life relationships influence social cognition and to unravel the role of underlying neural processing in shaping the development of relationships of differing valence over time.

## Electronic supplementary material

ESM 1 (DOCX 260 kb)
