# Supplementary material for: Friend versus foe: Neural correlates of prosocial decisions for liked and disliked peers
Source: Cogn Affect Behav Neurosci. 2018 Jan 9;18(1):127–42. doi: 10.3758/s13415-017-0557-1 (PMC5823968; doi:10.3758/s13415-017-0557-1)
Supplement: Supplementary file 1 — (DOCX 260 kb) [file 13415_2017_557_MOESM1_ESM.docx]

**Supplementary Materials**

**Distribution of behavior and beta estimates**

We did not exclude participants based on a minimum number of responses in a specific condition in the analyses. Table S1 provides an overview of how many participants had more than zero to five trials in the contrasts discussed in the Results section of the manuscript. To examine the robustness of our findings, we reran the whole-brain contrasts Friend Prosocial > Disliked Peer Prosocial, Friend Prosocial > Unfamiliar Peer Prosocial, and Disliked Peer Selfish > Friend Selfish in which we excluded participants with only one trial. These results are described in the manuscript. In Fig. S1 we show the distribution of beta estimates from the clusters obtained in the Friend Prosocial > Disliked Peer Prosocial and Disliked Peer Selfish > Friend Selfish *t* contrasts for each of the 27 participants. Importantly, Fig. S1 shows that there were no outliers that could have driven our findings where all participants are included.

**Brain regions of activation during interactions with friends and disliked peers**

First, we examined the neural underpinnings of decision making for friends and disliked peers regardless of behavior. The whole-brain one-sample *t* test of Friend > Disliked Peer (controlling for the frequency of prosocial behavior) did not yield significant clusters of brain activation. The Friend > Unfamiliar Peer contrast resulted in activation in the right inferior parietal lobule (IPL) extending toward the angular gyrus, and left IPL extending toward the superior parietal lobule. These brain regions are referred to as pTPJ–IPL. The whole-brain *t* contrasts of Disliked Peer > Friend, Disliked Peer > Unfamiliar, Friend > Neutral Peer, and Disliked Peer > Neutral Peer did not result in significant clusters of activity. The fact that there were no differences in neural activation for friends and disliked peers in the Friend > Disliked Peer and the reverse contrast were unexpected. Together with the results showing neural differences in the Friend Prosocial > Disliked Peer Prosocial and Disliked Peer Selfish > Friend Selfish contrasts, our findings suggest that at the neural level it is not the valence of the relationship with the interaction partner per se that affects the underlying neural processes differently, but rather the specific behavior for that interaction partner.

Next, we examined the neural correlates of prosocial and selfish decisions during interactions with friends and disliked peers. The whole-brain one-sample *t* test for prosocial decisions for friends compared to neutral peers (Friend Prosocial > Neutral Peer Prosocial) controlled for the frequency of prosocial choices yielded heightened activation in the left inferior frontal gyrus (*n* = 24). The Friend Selfish > Neutral Peer Selfish contrast did not result in significant neural activation. The Disliked Peer Prosocial > Neutral Peer Prosocial, and Disliked Peer Selfish > Neutral Peer Selfish also did not yield significant increased brain activation.

**Brain regions of activation during decisions for neutral peers**

We examined the neural correlates of decision making for neutral peers regardless of behavior. The Neutral Peer > Friend and Neutral Peer > Disliked Peer *t* contrasts did not yield significant activation clusters.

Next, we examined the neural correlates of prosocial and selfish decisions during interactions with neutral peers. The Neutral Peer Selfish > Friend Selfish contrast yielded activation in the left amygdala extending toward the temporal pole (*n* = 26). The Neutral Peer Prosocial > Friend Prosocial, Neutral Peer Prosocial > Disliked Peer Prosocial, and Neutral Peer Selfish > Disliked Peer Selfish contrasts did not yield significant heightened neural activation.

**Brain regions of activation during decisions for unfamiliar peers**

We examined the neural underpinnings of decision-making for unfamiliar peers regardless of behavior. The Unfamiliar Peer > Disliked Peer contrast showed activation in the dorsal anterior cingulate cortex and the left lateral prefrontal cortex. The Unfamiliar Peer > Friend did not yield significant activation clusters.

Next, we conducted *t* tests to examine neural activation for unfamiliar peers during prosocial and selfish choices. The Unfamiliar Peer Prosocial > Friend Prosocial, Unfamiliar Peer Prosocial > Disliked Peer Prosocial, Unfamiliar Peer Selfish > Friend Selfish, Unfamiliar Peer Selfish > Disliked Peer Selfish contrasts did not yield significant heightened brain activation for unfamiliar peers. Table S2 provides a summary of all the results.

**Brain and behavior links for friends and disliked peers versus neutral peers**

The percentage of prosocial choices for friends minus neutral peers in the Friend > Neutral Peer contrast did not result in any significant or positive relations with brain activity. To investigate the brain and behavior links during interactions with disliked peers, we included the difference scores of the percentage of prosocial choices for disliked peers minus neutral peers as a regressor in the Disliked Peer > Neutral Peer *t* contrast. This showed a negative correlation between the frequency of prosocial choices for disliked peers minus neutral peers and an activation cluster in the left inferior frontal gyrus. Correlation coefficients indicated that this negative relation was driven by individual differences in prosocial choices for disliked peers rather than for neutral peers (correlation coefficients of the relation between the parameter estimates of the interior frontal gyrus and the percentage of prosocial choices for disliked peers and neutral peers separately were −.57 and .08, respectively). This analysis did not yield a positive correlation between brain and behavior links for disliked peers versus neutral peers. Table S3 provides a detailed overview of these results.

**Table S1** Number of participants with more than zero to five trials

|  | *n* > 0 | *n* > 1 | *n* > 2 | *n* > 3 | *n* > 4 | *n* > 5 |
| --- | --- | --- | --- | --- | --- | --- |
| Friend Prosocial > Disliked Peer Prosocial | 23 | 18 | 17 | 14 | 14 | 11 |
| Friend Prosocial > Unfamiliar Peer Prosocial | 23 | 23 | 22 | 20 | 19 | 19 |
| Disliked Peer Selfish > Friend Selfish | 26 | 24 | 23 | 22 | 21 | 20 |

**Table S2** Regions of neural activation for friends and unfamiliar peers whole-brain contrasts controlling for frequency of behavior of interest

| Brain region | L/R | Voxels | *z* | MNI coordinates |
| --- | --- | --- | --- | --- |
|  |  |  |  | *x, y, z* |
| **Friend > Unfamiliar Peer** |  |  |  |  |
| pTPJ–IPL | R | 399 | 4.26 | 30 −54 36 |
|  |  |  | 3.9 | 42 −60 51 |
|  |  |  | 3.37 | 42 −54 39 |
| pTPJ–IPL | L | 196 | 3.77 | −48 −51 42 |
|  |  |  | 3.36 | −24 −54 42 |
|  |  |  | 2.97 | −36 −39 33 |
|  | | |  |  |
| Dorsal anterior cingulate cortex | – | 269 | 4.24 | −12 30 36 |
|  |  |  | 4.19 | 18 33 21 |
|  |  |  | 4 | −21 36 27 |
| Lateral prefontal cortex | L | 150 | 4.14 | −33 45 −9 |
|  |  |  | 3.67 | −18 57 −3 |
|  |  |  | 3.47 | −24 45 −3 |
| **Prosocial choices** | |  |  |  |
| *Friend > Neutral Peer* | | |  |  |
| Inferior frontal gyrus | L | 149 | 4.48 | −54 15 6 |
|  |  |  | 3.02 | −54 27 0 |
| **Selfish choices** | |  |  |  |
| *Neutral Peer > Friend* | | |  |  |
| Amygdala–fusiform gyrus–temporal pole | L | 205 | 3.9 | −24 −3 −24 |
|  |  |  | 3.79 | −30 0 −33 |
|  |  |  | 3.54 | −36 9 −33 |

*Note.* Analyses are conducted using FWE cluster correction at *p* < .05 with a cluster-forming threshold of *p* < .005

**Table S3** Regions of neural activation of correlations between prosocial choices and whole-brain *t* contrasts

| Brain region | L/R | Voxels | *z* | MNI coordinates |
| --- | --- | --- | --- | --- |
|  |  |  |  | *x, y, z* |
| **Disliked peer > Neutral Peer** | |  |  |  |
| *Mean prosocial choices for disliked peers-neutral peers as negative regressor* | | | | |
| Inferior frontal gyrus | L | 119 | 4.33 | −54 9 18 |
|  |  |  | 3.13 | −54 0 21 |
|  |  |  | 2.92 | −51 30 18 |

*Note.* Analyses are conducted using FWE cluster correction at *p* < .05 with a cluster-forming threshold of *p* < .005


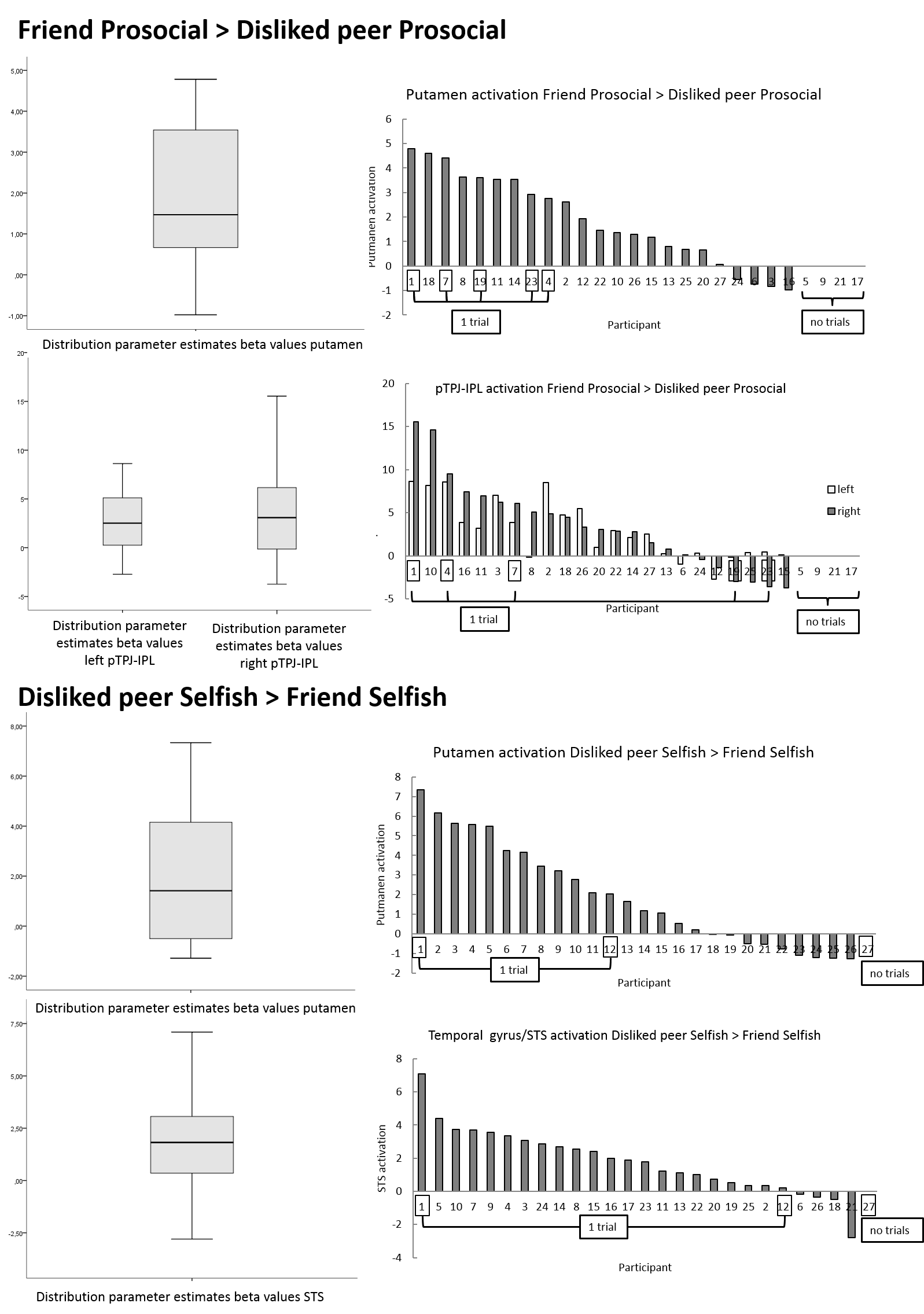


**Fig. S1** Distribution of activation clusters from the Friend Prosocial > Disliked Peer Prosocial and Disliked Peer Selfish > Friend Selfish *t* contrasts for each of the 27 participants
